# Supplementary material for: At-RS31 orchestrates hierarchical cross-regulation of splicing factors and integrates alternative splicing with TOR-ABA pathways
Source: bioRxiv. 2024 Dec 7:2024.12.04.626797. Preprint. [Version 1] doi: 10.1101/2024.12.04.626797 (PMC11643119; doi:10.1101/2024.12.04.626797)
Supplement: 1 [file NIHPP2024.12.04.626797v1-supplement-1.pdf]

bioRxiv preprint doi: https://doi.org/10.1101/2024.12.04.626797; this version posted December 7, 2024. The copyright holder for this preprint (which was not certified by peer review) is the author/funder, who has granted bioRxiv a license to display the preprint in perpetuity. It is made available under aCC-BY 4.0 International license.

SUPPORTING INFORMATION

SUPPLEMENTAL FIGURES

Fig. S1

At-RS31-GFP fusion protein expressed in transgenic plants used in the iCLIP

Fig. S2

GST-tagged At-RS31 fusion proteins used for RNAcompete

Fig. S3

Immunopurification of At-RS31 protein–RNA complexes from UV crosslinked *RS31::RS31-GFP* and *35S::GFP* plants and preparation of iCLIP libraries

Fig. S4

Genome-wide distribution of crosslink sites

Fig. S5

Sequence logo of At-RS31 binding sites enriched upstream of 5' splice sites

Fig. S6

RNAcompete analysis of the At-RS31 protein

Fig. S7

RT-PCR analyses of differential alternative splicing events in genes with At-RS31 binding sites identified by iCLIP

bioRxiv preprint doi: https://doi.org/10.1101/2024.12.04.626797; this version posted December 7, 2024. The copyright holder for this preprint (which was not certified by peer review) is the author/funder, who has granted bioRxiv a license to display the preprint in perpetuity. It is made available under aCC-BY 4.0 International license.

Fig. S8

RT-PCR analyses of differential alternative splicing in genes encoding RNA binding proteins and splicing factors, including SR proteins

Fig. S9

Examples of At-RS31 and TOR pathway shared targets

SUPPLEMENTARY TABLES

Table S1

Oligonucleotides used in this study

Table S2

iCLIP read statistics

Table S3

At-RS31 iCLIP binding site coordinates

Table S4

At-RS31 iCLIP target transcripts

Table S5

Functional enrichment analysis

Table S6

Distances from transcription start sites to At-RS31 binding sites

Table S7

Regions upstream of 5' splice sites containing At-RS31 binding sites

Table S8

Differential alternative splicing analysis for At-RS31 mutant and overexpression plants

Table S9

Differential gene expression analysis for At-RS31 mutant and overexpression plants

Table S10

Transcription factors modulated by At-RS31

bioRxiv preprint doi: https://doi.org/10.1101/2024.12.04.626797; this version posted December 7, 2024. The copyright holder for this preprint (which was not certified by peer review) is the author/funder, who has granted bioRxiv a license to display the preprint in perpetuity. It is made available under aCC-BY 4.0 International license.

Table S11

Shared targets of At-RS31 and the TOR pathway

Table S12

At-RS31 in abscisic acid metabolism and signaling
